# Extended analysis on peripheral blood cytokines correlated with hepatitis B virus viral load in chronically infected patients – a systematic review and meta-analysis

**DOI:** 10.3389/fmed.2024.1429926

**Published:** 2024-07-31

**Authors:** Marina Manea, Ion Mărunțelu, Ileana Constantinescu

**Affiliations:** ^1^Immunology and Transplant Immunology, University of Medicine and Pharmacy “Carol Davila”, Bucharest, Romania; ^2^Center of Immunogenetics and Virology, Fundeni Clinical Institute, Bucharest, Romania

**Keywords:** cytokine, hepatitis B virus, viral load, systematic review, meta-analysis

## Abstract

**Background:**

Hepatitis B Virus (HBV) can affect life quality. Monitoring and understanding the fluctuations of the HBV level of viremia related to the intricate immune activity of the host helps in the development of new treatment strategies and evaluation patterns. This meta-analysis presents the correlations between cytokines and the level of viremia in chronic HBV patients for a better comprehension of the immune mechanisms behind this infection.

**Methods:**

We used PRISMA guidelines for this meta-analysis. The databases assessed were PUBMED, WEB OF SCIENCE, SCOPUS, and Cochrane Library. ZOTERO and PlotDigitizer helped the systematic research process. We extracted information related to the correlations between cytokines and the HBV-DNA level. Effect measures included comparisons between standardized mean differences and correlation coefficients. We evaluated retrieved articles with the Newcastle-Ottawa Quality Assessment Scale (NOS). The R 4.2.2 software displayed the statistical calculation and graphical representations.

**Results:**

From 58,169 records, we extracted 16 articles with 32 different cytokine determinations. The main interleukins included in detection panels were IL-10 and IL-21. The meta-correlation analysis comprised 1,199 chronic HBV patients. The standardized mean difference between cytokine levels in HBV patients and healthy controls was 0.82 (95% CI = [−0.19, 1.84], *p* = 0.11). We observed a significant, fair, pooled correlation coefficient between IL-10, IL-9, and the viral load (*r* = 0.52, 95% CI = [0.19, 0.85]).

**Conclusion:**

This meta-analysis brings novelty because it gives a first rigorous systematic look at multiple studies with many cytokines. Our research approaches a debatable issue and gives a possible solution for settling controversies. Future studies can arise towards understanding the immune disruption in HBV and the development of new, improved assays for prognosis.

## 1 Introduction

According to the World Health Organization (WHO), chronic infection with hepatitis B virus (HBV) is a worldwide increasingly mortal threat, with a high prevalence in Africa, some parts of Asia, Eastern Mediterranean, and Western Pacific countries. The most worrying event is the progression of the illness toward hepatocellular carcinoma (HCC), frequently found in Southeast Asia (1). To prevent this unfavorable outcome of HBV, the discovery of a cure becomes a desired goal for the scientific environment (2).

The deoxyribonucleic acid (DNA) of HBV is considered by some researchers a key element in the viral replication cycle. From its initial relaxed circular pattern, HBV-DNA converts into various forms, including a covalently closed circular DNA (cccDNA) (3). The latter persists in the host cell nucleus and it resembles a small chromosome after the addition of a histone complex (4). From the cccDNA several viral RNAs are transcribed. After the reverse transcription of pre-genomic RNA, a relaxed circular DNA (rcDNA) or a double stranded DNA are formed. Consequently, viral DNA either leaves the hepatocyte in new virions, or returns to the nucleus to replenish the pool of cccDNA (3).

Studies claim that the HBV-DNA quantification shows the viral activity in chronically infected patients (5). However, as Mak et al. (6) emphasize, the serum detection of viral DNA cannot directly establish the levels of those viral nucleic acids that remain silent in hepatocytes. Studies mention that these remnants of genetic material can trigger HCC (7–9). Some authors have tried to quantify the levels of cccDNA through various PCR techniques, but their efforts have concluded in an intricate and not fully developed protocol. Another problem is the need for invasive liver biopsy samples for cccDNA quantification (10). This problem could disappear by finding better predictors for the fluctuations of viral DNA levels inside the infected host. Some authors link cytokines to the HBV-DNA levels (11).

Scientists define cytokines as inhibitory or stimulatory elements on immune cells (12, 13). Studies establish the main categories of cytokines as different types of interleukin (IL), tumor necrosis factor-alpha (TNF-α), transforming growth factor-beta (TGF-β), and interferon (IFN) (13).

Other researchers linked several genetic variations and the ability of cytokines to participate in viral clearance- this is the case of the allelic polymorphisms -148C, +8925G, +13925C for the IL-18 gene, -592CA for the IL-10 gene, or -863A for the TNF-α gene (11). Studies also provide the example of IL-2, IL-4, and IL-21, which interfere with the expansion of T lymphocytes in chronic HBV (12). Scientists also correlated HBV viral loads with immune cell counts, so their activity should be broadly analyzed (14). Recent experiments illustrate a combined effect of cytokines, for which reason they could be assessed together (15). Other studies show that Toll receptor agonists, as new potential therapies, enhance interleukin production and reduce the levels of viral proteins and of HBV-DNA (16).

Regarding the common usage of cytokines as biomarkers in chronic HBV, potential assays exist for their detection. As an example, researchers have mentioned inexpensive techniques for IL-6, IL-1β, IL-3, IL-8, and TNF-α (17). Although the evaluation of cytokines is not a well-implemented routine for chronic HBV patients, this might demonstrate its benefits. Studies emphasize the prediction qualities of IL-6 (18) and IL-2 (19). The latter, together with cytokines such as IL-6, IL-4, IL-1β, and IL-17A can also anticipate treatment response for nucleotide/nucleoside analogs (NA) and pegylated interferon (PegIFN) (20). The level of HBV-DNA has already been used as a biomarker for disease evolution and treatment effectiveness (21), so a proven association between viremia and cytokines could potentially increase the prediction capacity of the two.

Some authors show that IL-4 correlates with HBV viral load (12). Researchers included this interleukin and other cytokines in their research. They found no correlations between cytokines and the levels of HBV-DNA (22). Opinions related to this topic are divided.

Considering all the discoveries presented above, cytokine studies in chronic HBV patients might change the upcoming evaluation techniques. However, their fluctuations in patients’ sera and their correlations with the levels of HBV-DNA need further examination for a definitive conclusion.

Our study aims to perform a meta-analysis of the cytokine level variations in chronic HBV patients. We wanted to assess potential correlations between cytokines and the levels of HBV-DNA. To our knowledge, this is the first meta-analysis on this theme performed on many cytokines and patients. This work intends to improve understanding of the precise correlations between cytokines and viremia and highlight potential gaps in knowledge. The effort is important to emphasize interesting novel tools for prognosis and therapy and suggest further themes for study.

## 2 Methods

### 2.1 Databases and search method

This meta-analysis was thought after the Preferred Reporting Items for Systematic Reviews and Meta-analyses (PRISMA) Statement (23). The protocol synthesis can be found in Supplementary Table S1. PUBMED, WEB OF SCIENCE, SCOPUS, and Cochrane Library were the four databases used. Data included all entries from inception until March 7th, 2024. The manual search was performed independently by two authors (MM and IM). The third researcher (IC) supervised the process and solved any debatable issues. Chronic HBV patients were a central part of this study, together with the comparisons between cytokine values in subjects with different levels of viremia. We also searched correlations between viral loads and cytokines. The words “chronic hepatitis B”, “chronic HBV infection,” “viral hepatitis B,” “HBV infection,” “HBV,” “VHB,” “hepatitis B virus,” “low viremia,” “low HBV DNA,” “low viral DNA,” “HBV DNA,” “high viremia,” “high HBV DNA,” “high viral DNA,” “Il,” “IL,” “interleukin,” “cytokine,” “cytokine assay,” “interleukin assay” composed our search strategy focused on retrieving articles in all databases. We used the same general terms for all databases, and we did not limit the field of search. For further information, please consult Supplementary Table S2.

### 2.2 Selection criteria

The peripheral blood cytokine values found in chronic HBV patients were the main elements of our search. The central topic followed was cytokine quantification for different levels of HBV-DNA. The manual data extraction was performed independently by two authors (MM and IM). Debatable issues were solved by discussion with the third researcher (IC). We also extracted data related to the correlation between cytokines and viremia. Without these items, records were not relevant. We included only English-written articles with full-text availability. The exclusion process also meant eliminating all abstracts, pre-prints, proceedings, conference recordings, reviews, meta-analyses, systematic reviews, book chapters, surveys, editorials, notes, letters, and commentaries. We did not include the retracted or duplicate papers.

### 2.3 Data extraction and quality assessment method

MM and IM independently assessed and extracted all data. Discussion and third-party evaluation by IC solved every difference in opinion. An in-house conceived form gathered information, and the authors developed tables to summarize data. The variables assessed included authorship information, comparison groups, detection methods, and cytokine levels. Correlation coefficients between interleukins and the levels of HBV-DNA were also extracted for our meta-analysis. Some items were taken from figures using PlotDigitizer (24). ZOTERO (25) helped in the elimination of duplicate articles. We used the Newcastle-Ottawa Quality Assessment Scale (NOS) (26) for evaluating the risk of bias and study quality. Some articles were cross-sectional, so they were assessed using a modified NOS scale that was previously used by other authors (27, 28). According to this tool, we evaluated studies after the sample selection, comparability of groups, and exposure/outcome assessment. All articles ranked with more than 7 stars were categorized as having low bias risk and high-quality (26, 29).

### 2.4 Statistics

We used R 4.2.2 Software (R Foundation for Statistical Computing, Vienna, Austria) for statistical analysis (30). A random-effect method and an inverse variance weighting model assessed the interleukin variations between the different categories of patients. Therefore, we used the indications of Borensen et al. (31) for a standardized mean difference calculation. According to the same authors, unlike fixed-effect methods, random-effect calculations take into consideration several reasons for study heterogeneity, so that is why we decided to use them (31). The Luo et al. (32) methodology was the basis for unavailable mean retrieval. A Shi et al. (33) model was the mathematical resource for standard deviations. Correlations between cytokine values and the HBV-DNA levels were assessed using Fisher’s z-transformed correlations, according to Cooper et al. (34). We used the *I^2^* statistic, described by Higgins et al. (35), and 75% was the limit for high heterogeneity. The Guddat et al. (36) explanations guided our forest plot assembly. Sterne and Egger (37) had envisioned the funnel plot method that we used for further bias assessment. The Egger et al. (38) test analyzed asymmetry in funnel graphics. Sensitivity analysis and subgroup displays were also used, as recommended by Egger et al. (39). The guide for significance in all our statistical calculations was a *p*-value under 0.05.

## 3 Results

### 3.1 The article selection

**Figure 1:**
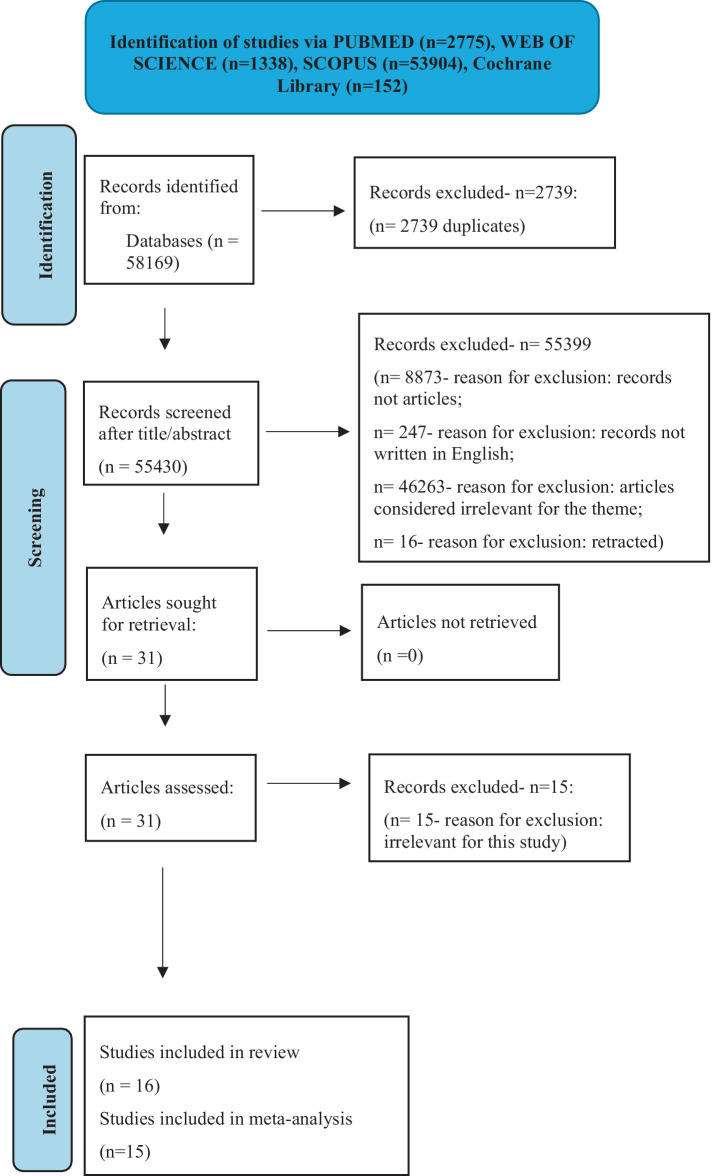
Study selection process (*n* = number) – figure adapted after PRISMA guidelines (23).

We retrieved 58,169 records from four databases (PUBMED- 2775 records, WEB OF SCIENCE- 1338 records, SCOPUS- 53904 records, and Cochrane Library- 152 records). The elimination process started with the exclusion of duplicates. Research other than article-based was also left aside together with non-English papers and retracted records. 46,263 articles were irrelevant for this study because they were not theme-related. We continued to eliminate another 15 records from the remaining studies because their authors did not perform calculations for the data needed in our study. The final systematic review included 16 articles. One of them could not be part of the final meta-analysis. The reason was that it contained poor data presentation with missing patients, that questioned its rigor. For the best quality of our meta-analysis, we decided to include only the most rigorous papers. Figure 1 shows every step of the extraction process.

### 3.2 Main characteristics and quality assessment of studies

As presented above, we selected 16 articles for systematic analysis, written between 2011 (40) and 2023 (41). They contained 32 different cytokine analyses. Most studies were Chinese (42–52). 8 articles were cohort-based (40, 42, 44, 46, 49–51, 53), 6 were case–control studies (41, 43, 45, 47, 48, 52), and 2 were cross-sectional (54, 55). The total number of participants was 2,249, and more than half were chronic hepatitis B patients (CHB). Only two of the retrieved studies analyzed cytokines from plasma (44, 50) using Elisa techniques. All the other articles collected data about cytokine levels from serum samples, mainly with Elisa methods (41, 43, 45–49, 52, 54). IL-10 was the most studied interleukin from all panels (40, 42, 48, 51, 53), together with IL-21 (43, 44, 51–53). The overall quality of the retrieved studies was considered high because most had an NOS score equal to or higher than 7 (26–28). One (51) quality score was lower than 7, so the article was eliminated because we feared bias. The potential risks of error identified at this phase were due to the original authors’ faults in the evaluation or follow-up of patients and healthy individuals.

Table 1 depicts the main description of the selected articles. One study (51) could not be included in the meta-analysis because of poor original data presentation. In this paper, the authors found a statistically significant association between two plasma cytokines (MCPI and IL-8) and HBV viral loads. The entire study collected data from 347 patients, but the final statistics contained information from only 168 individuals. The methodology had also issues in data analysis (51).

**Table 1:** General study characteristics of the articles selected for systematic review.

Study (Author, Year)	Country	Study type	Study groups	Method for cytokine detection	Cytokines studied	NOS
Park et al. (40)	Korea	Cohort	27 CHB 20 HC	Cytokine array (from serum)	IL-1α, IL-1β, IL-2, IL-4, IL-6, IL-8, IL-10, VEGF, IFN-γ, MCPI, EGF, TNF-α	7
Li et al. (42)	China	Cohort	40 CHB 30 HC	Cytokine bead array (for serum IL-2 and IL-10); Elisa (for serum IL-37)	IL-2, IL-10, IL-37	8
Okuhara et al. (53)	Japan	Cohort	48 CHB 10 HC	Luminex (from serum)	IL-2, IL-6, IL-10, IL-12p70, IL-21, IL-22	8
Li et al. (43)	China	Case–control	102 CHB 25 HC	Elisa (from serum)	IL-21	7
Zhong et al. (44)	China	Cohort	91 CHB 30 HC	Elisa (from plasma)	IL-7, IL-21	9
Cheng et al. (45)	China	Case–control	79 CHB 105 HC	Elisa (from serum)	IL-34	8
Shao et al. (46)	China	Cohort	37 CHB 24 ASC 20 HC	Elisa (from serum)	IL-35	9
Cheng et al. (47)	China	Case–control	72 CHB 41 HC	Elisa (from serum)	IL-35	8
Cui et al. (48)	China	Case–control	22 CHB 16 HC	Elisa (from serum)	IL-9, IL-10	8
Metanat et al. (54)	Iran	Cross-sectional	143 CHB	Elisa (from serum)	IL-17	7*
Wiegand et al. (55)	Germany	Cross-sectional	333 HBV infected	Luminex (from serum)	IL-17, TGF-β1, TGF-β2, TGF-β3, TNF-α, IL-12, IL-16	7*
Luo et al. (49)	China	Cohort	30 CHB 28 HC	Elisa (from serum)	IL-26, IL-17	9
Yang et al. (50)	China	Cohort	102 CHB	Elisa (from plasma)	IL-12	7
Ren et al. (51)	China	Cohort	347 CHB 20 HC	Luminex (from serum)	MCPI, FGF-2, IFN-α2, IFN-γ, IL-1β, IL-2, IL-6, IL-8, IL-10, IL-17, IL-21, TNF-α, VEGF-α, IP-10	6
Zhou et al. (52)	China	Case–control	142 CHB 20 HC	Elisa (from serum)	IL-12, IL-18, IL-21	8
Elbrolosy et al. (41)	Egypt	Case–control	185 CHB 60 HC	Elisa (from serum)	IL-6	8

CHB, chronic hepatitis B; HBV, hepatitis B virus; HC, healthy controls; ASC, asymptomatic carrier; IL, interleukin; IL-1α,β, interleukin 1 alpha, beta; TGF-β1,2,3, transforming growth factor beta 1,2,3; TNF, tumor necrosis factor; MCPI, monocyte chemotactic protein; FGF, fibroblast growth factor; IFN-α2, interferon alpha 2; IFN-γ, interferon gamma; VEGF, (α)-vascular endothelial growth factor (alpha); IP-10, interferon gamma inducible protein 10; NOS, Newcastle-Ottawa scale.^*^Studies that required adapted NOS (27, 28).

We conducted a meta-analysis of 6 articles for the assessment of cytokine levels in different types of HBV patients with several viremia loads (41, 42, 49, 52, 54, 55). These studies were presented in Supplementary Table S3. 9 records containing Spearman correlations (40, 41, 43, 45, 47–50, 53) were used for a separate meta-correlation analysis, and their main details were illustrated in Table 2. Two studies (44, 46) included different types of correlations (Pearson correlations), so our meta-analysis had to exclude them. Both studies were cohort-type and included Chinese patients. One study showed significant Pearson correlations between IL-7, IL-21, and the levels of HBV-DNA in CHB (44). The other found a significant correlation between IL-35 and viral loads in chronic HBV patients (46).

**Table 2:** Correlations found between cytokines and HBV viremia level H.

Study (Author, Year)	Country	Study type	Study group (patients)	Age-mean ± standard deviation (years)	Method for cytokine detection	Cytokine studied	Correlation between cytokines and viremia-r coefficient	NOS
Park et al. (40)	Korea	Cohort	27 CHB	NA	Cytokine array (from serum)	IL-1α	−0.15	7
						IL-1β	0.29	
						IL-2	0.09	
						IL-4	0.24	
						IL-6	0.27	
						IL-8	0.35	
						IL-10	0.6	
						VEGF	0.04	
						IFN-γ	−0.02	
						MCPI	0.17	
						EGF	0.09	
						TNF-α	0.31	
Okuhara et al. (53)	Japan	Cohort	48 CHB	54.55 ± 12.7	Luminex (from serum)	IL-2	0.08 0.01 0.1 0.06 0.08 0.17	8
						IL-6		
						IL-10		
						IL-12p70		
						IL-21		
						IL-22		
Li et al. (43)	China	Case–control	25 CHB-IC mild 25 CHB-IC moderate 25 CHB-IC severe	32.7 ± 3.2	Elisa (from serum)	IL-21	0.392	7
				36.2 ± 1.5				
				35.5 ± 2.5				
Zhong et al. (44)	China	Cohort	91 CHB	36.14 ± 11.16	Elisa (from plasma)	IL-7	−0.21* −0.36	9
						IL-21		
Cheng et al. (45)	China	Case–control	79 CHB	42.76 ± 38.46	Elisa (from serum)	IL-34	−0.25	8
Shao et al. (46)	China	Cohort	37 CHB 24 ASC	33.7 ± 10.1 26.9 ± 8.6	Elisa (from serum)	IL-35	0.31*	9
Cheng et al.(47)	China	Case–control	72 CHB	41 ± 38.54	Elisa (from serum)	IL-35	−0.28	8
Cui et al. (48)	China	Case–control	22 CHB	48.14 ± 10.34	Elisa (from serum)	IL-9	0.585 0.625	8
						IL-10		
Luo et al. (49)	China	Cohort	30 CHB	NA	Elisa (from serum)	IL-26	0.072	9
Yang et al. (50)	China	Cohort	102 CHB	37.95 ± 8.54	Elisa (from plasma)	IL-12	−0.147	7
Elbrolosy et al. (41)	Egypt	Case–control	65 inactive HBV infection 62 active CHB 58 cirrhosis	52.5 ± 11.8	Elisa (from serum)	IL-6	0.26 0.35 0.39	8
				55.3 ± 9.4				
				54.5 ± 11.7				

CHB, chronic hepatitis B; HBV, hepatitis B virus; IL, interleukin; IL-1α, β, interleukin 1 alpha, beta; VEGF, vascular endothelial growth factor; IFN, interferon; MCPI, monocyte chemotactic protein 1; EGF, epidermal growth factor; TNF-α, tumor necrosis factor alpha; IC, immuno-clearance phase; ASC, asymptomatic carrier; NA, not available.^*^The correlations used in this article are Pearson correlations.

### 3.3 Cytokine level differences between healthy subjects and HBV patients

**Figure 2:**
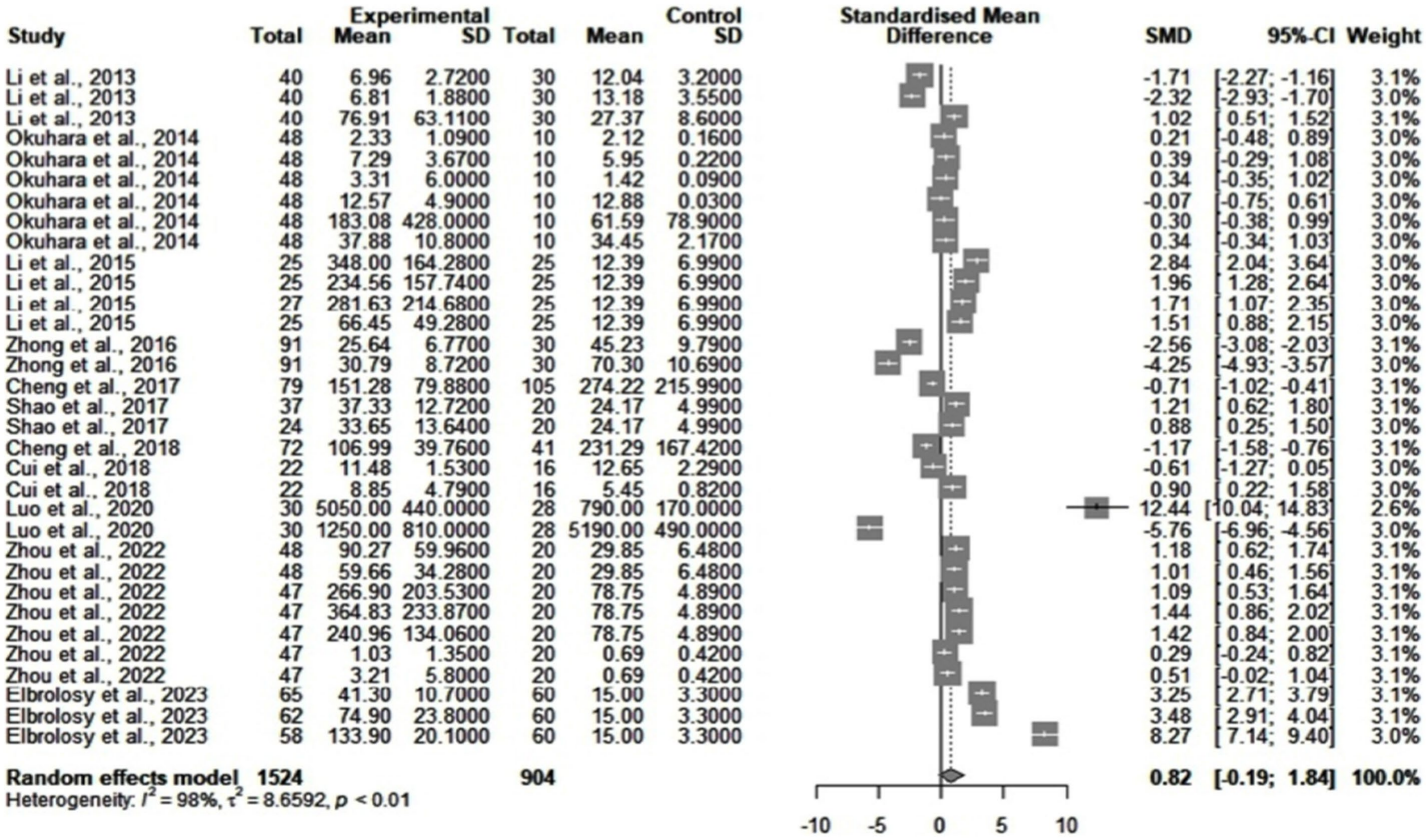
Differences between cytokine levels in healthy subjects (control group) and chronic HBV patients (experimental group).

Figure 2 shows the differences between cytokine values in chronic HBV patients and healthy subjects. The statistical analysis found significant heterogeneity among the studies (*I^2^* = 98%). We searched for publication bias using a funnel plot and the Egger’s test. The funnel plot showed no obvious asymmetry. Egger’s test did not identify publication bias. The standardized mean difference (SMD) between the two groups was 0.82 (95% CI = [−0.19, 1.84], *p* = 0.11). Subgroups were then drawn after the type of cytokine used, the detection method, and the sample size. Using meta-regression techniques, we found that one source of heterogeneity could come from the quantification of some IL, such as IL-26 (*p* < 0.0001) and IL-6 (*p* = 0.0073). However, after removing these studies, the remaining heterogeneity was still large (*I^2^* = 97%). An important element was also the small number of studies for each subgroup.

We further analyzed subgroups with more than two study results. The peripheral blood samples taken from patients had significantly higher levels of IL-21 by comparison to those drawn from healthy individuals (SMD = 0.92, *p* = 0.048). However, study heterogeneity was high (*I^2^* = 95%). We could not perform further analysis because of the small number of studies found (4 studies). IL-10 was lower in HBV patients (SMD = -0.85), but the result was nonsignificant (*p* = 0.2836) and with high heterogeneity (*I^2^* = 94%).

### 3.4 Meta-correlation results

**Figure 3:**
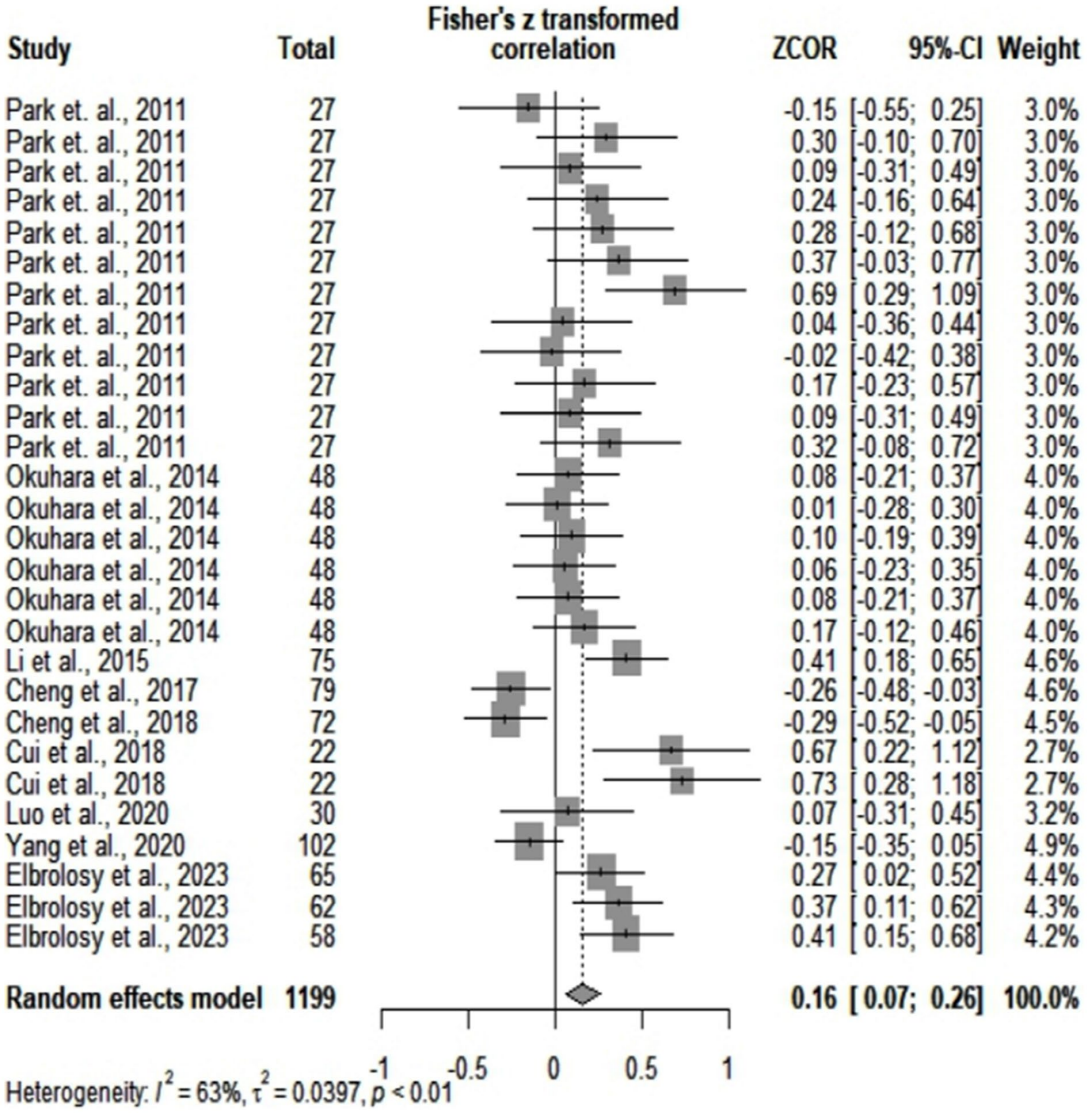
Results obtained from meta-correlation analysis.

**Figure 4:**
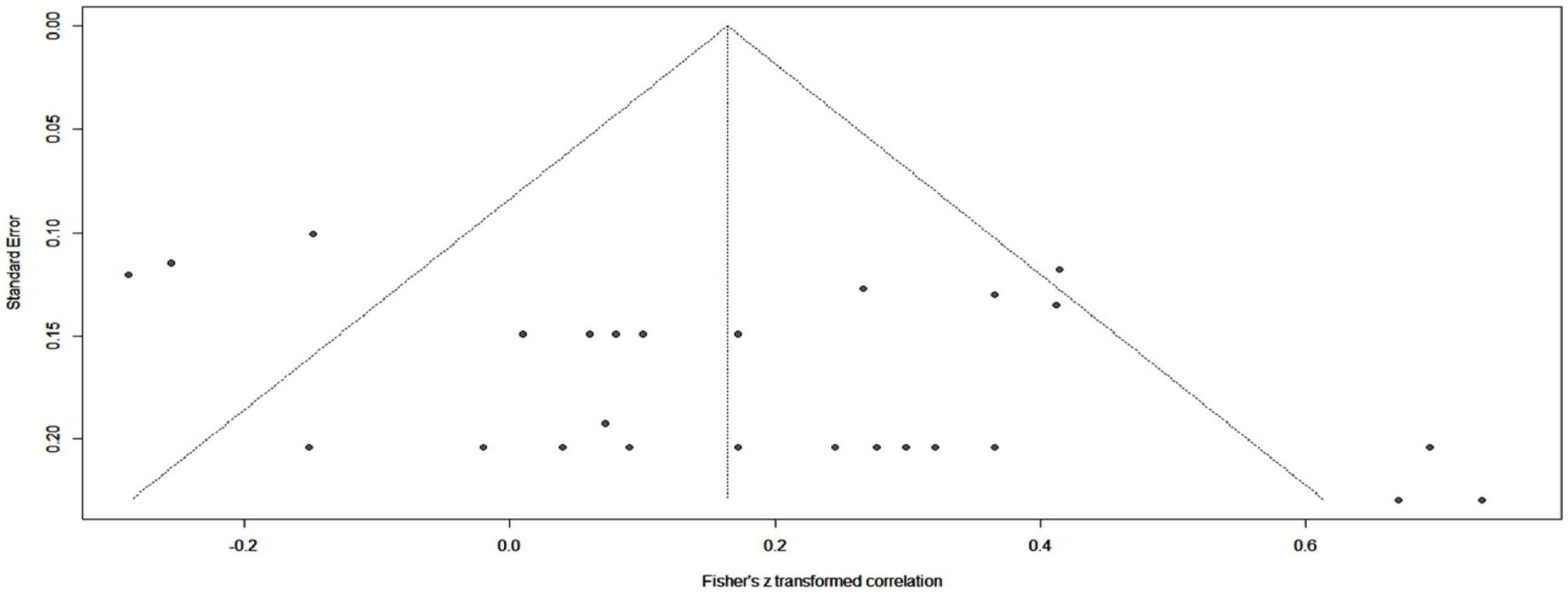
Funnel plot obtained from the meta-correlation analysis.

After statistically analyzing the 9 studies included in meta-correlation, we found a pooled correlation coefficient of 0.16 (*p* = 0.0009) between all cytokine values and the levels of viremia. The overall heterogeneity was moderate (*I^2^* = 63%), according to Higgins et al. (35). Figures 3, 4 depict the graphic representations of these results with a forest plot and a funnel plot. The latter had no obvious asymmetry, and the trim-and-fill test showed no obvious publication bias (*p* = 0.82). Meta-regression performed on age, sample size, and IL-type subgroups suggested that a large amount of heterogeneity could be explained by studies that included IL-34, IL-12, and IL-35. After removing these, the residual heterogeneity was not significant (*I^2^* = 29%, *p* = 0.09), and the pooled correlation coefficient was 0.23 (*p* < 0.0001).

**Figure 5:**
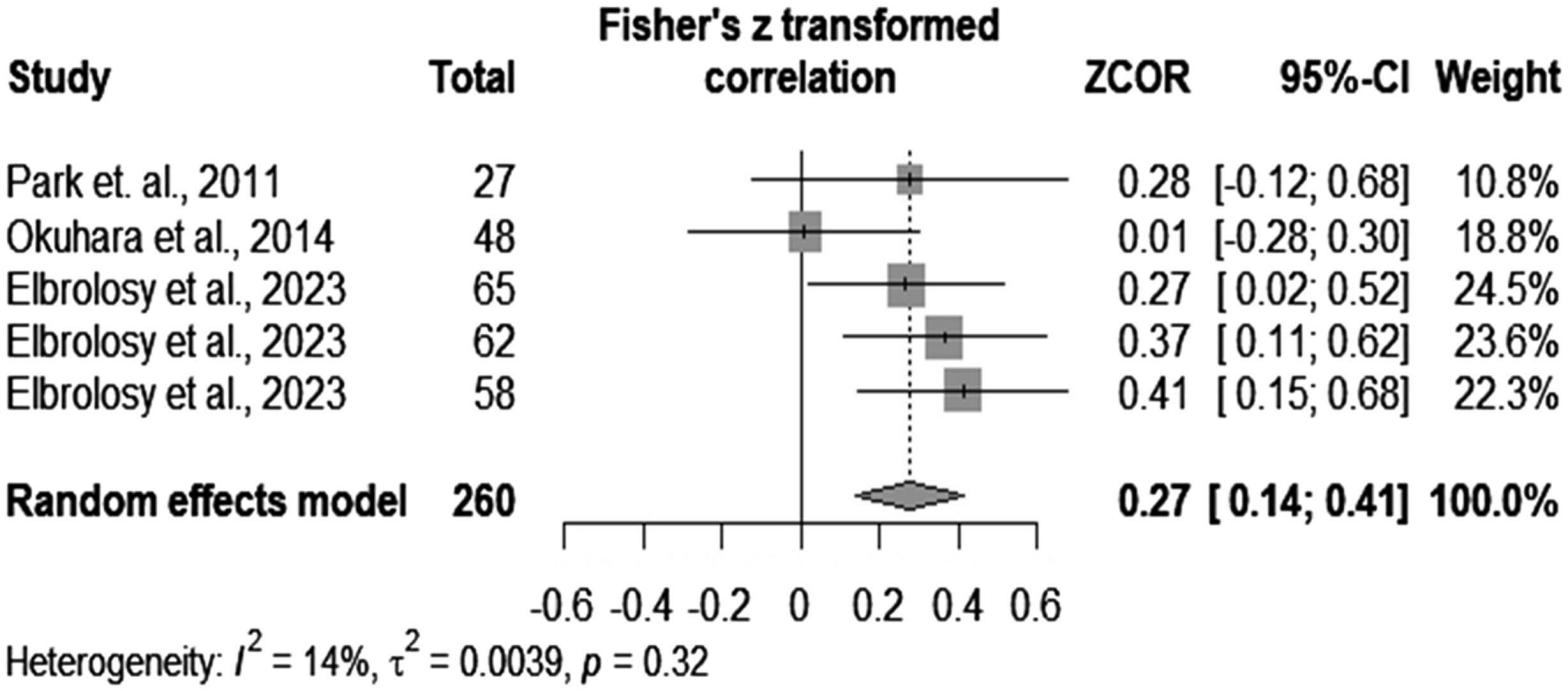
IL-6 meta-correlation analysis.

**Figure 6:**
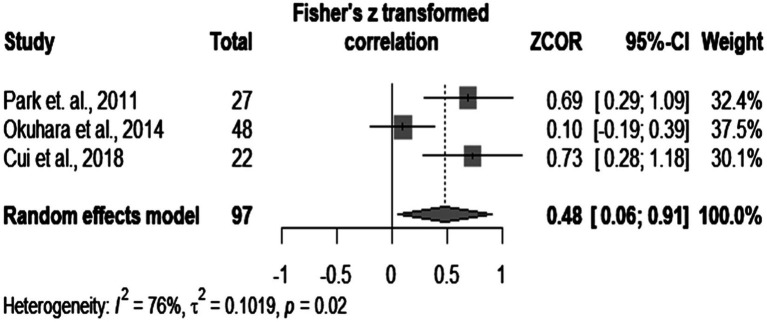
IL-10 meta-correlation analysis.

Further subgroup analysis showed that IL-6 had a pooled correlation coefficient of 0.27 (*p* < 0.0001), with no significant heterogeneity (results depicted in Figure 5). The correlation coefficient of IL-10 was 0.48 (*p* = 0.02), but significant heterogeneity between studies was found (*I^2^* = 76%, *p* = 0.02). These numbers can be visualized in Figure 6.

**Figure 7:**
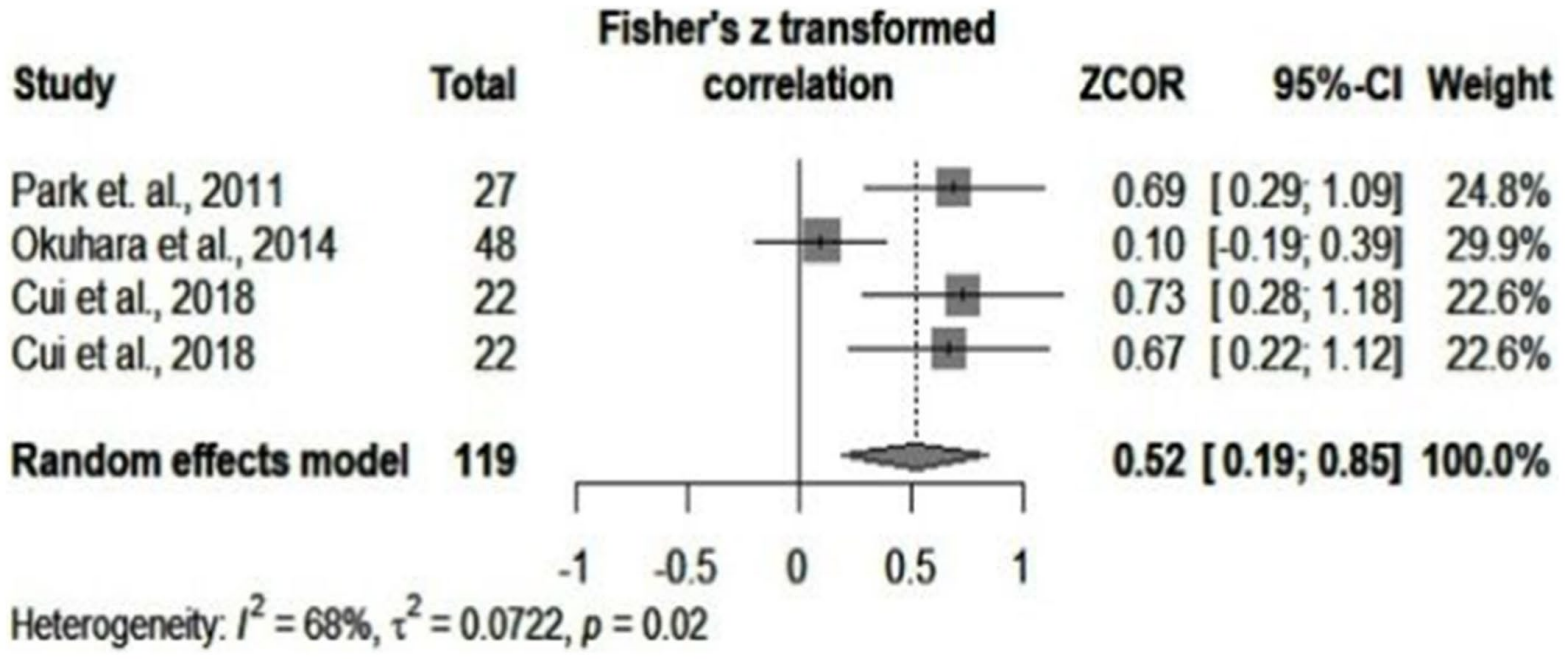
IL-10 and IL-9 meta-correlation analysis.

We assessed the studies with the highest correlation values between cytokines and the levels of viremia to see if a higher degree of association could be significant. Therefore, a pooled correlation coefficient of 0.52 (*p* = 0.002) was identified in the meta-correlation of IL-10 and IL-9. However, this also involved a moderate degree of heterogeneity (*I^2^* = 68%, *p* = 0.02). Figure 7 depicts this result. By adding IL-8, we observed a small decrease in the correlation coefficient to 0.48 and a lower percentage of heterogeneity. However, the number of studies taken into consideration was small.

### 3.5 Cytokine levels among patients in different HBV-DNA categories

Patient viremia levels were used to categorize the retrieved studies. A first comparison was performed between the groups of interest encompassing patients with the levels of HBV-DNA more or less than 2000 IU/mL (41, 54, 55). We chose this value because it represents a classical level included in guidelines that separates moderate viral loads from low ones (6). We wanted to assess the cytokine levels in high HBV-DNA patients, so we compared subjects with levels of viremia higher or lower than 10,000 IU/mL (42, 52), 100,000 IU/mL (49, 52), and 10,000,000 IU/mL (52, 54). The only significant results were obtained in articles that quantified HBV-DNA according to the 2000 IU/mL and 10,000,000 IU/mL criteria.

An increase in the cytokine levels was observed when subjects with levels of viremia higher and lower than 2000 IU/mL were compared (SMD = 0.52, *p* = 0.0019). The analyzed cytokines were IL-17, TGF-β1, TGF-β2, TGF-β3, TNF-α, IL-12, IL-16, and IL-6. There was significant heterogeneity observed (*I^2^* = 85.7%, *p* < 0.0001). We observed asymmetry on the funnel plot. Egger’s test (37) identified significant publication bias (*p* = 0.0178). Heterogeneity was also observed between groups when they were divided according to the type of cytokine analyzed (*p* < 0.0001). Further analysis could not be performed because of the small number of studies involved.

For patients with levels of viremia higher or lower than 10,000 IU/mL, we observed a funnel plot asymmetry, but Egger’s test did not show significant publication bias.

The comparison between the categories of patients with levels of viremia higher and lower than 10,000,000 IU/mL presented a significant decrease in cytokine levels (SMD = −0.47, *p* = 0.0063), with moderate heterogeneity (*I^2^* = 43.5%, *p* = 0.17). The differences between the subgroups selected after the types of cytokines were not significant. However, only three cytokines (IL-17, IL-18, IL-12) were assessed. The total number of studies was also small, thus limiting further analysis.

## 4 Discussion

Chronic HBV infection is a source of concern because of its worldwide persistence (1) and the need for its elimination (2). Researchers report that HBV-DNA quantification has a major importance in the evaluation and prognosis of a patient (5). According to some, the viral load can also contribute to establishing a therapeutic response (10, 56). Immune biomarkers are identified as another potential field of research in HBV chronic infections (19, 20, 56). This current meta-analysis reunites the two areas of interest in the first documented effort to systematically summarize the potential correlations between the levels of HBV-DNA and of cytokines.

### 4.1 The main findings of our study

The overall quality of retrieved studies was high, with 2,249 participants and 32 cytokines. This is, from our knowledge, the first large-scale analysis that included the assessment of cytokine in HBV patients.

By comparison to the normal controls, the cytokine levels seemed to increase (SMD = 0.82) in HBV patients, but with high heterogeneity. As studies depict, this could be explained by intricate molecular pathways (57–59). Therefore, authors show that HBV can activate various pathways, such as Phosphatidylinositol 3-kinase-AKT serine/threonine kinase (P13K–AKT), Janus Kinase-Signal transducer and activator of transcription 3 (Jak-STAT3), or Rat sarcoma virus gene-Rapidly accelerated fibrosarcoma (RAS–RAF) (57). This leads to inflammation, with an increase of several cytokines (IL-10, IL-4, IL-12, and TNF-α) (57) and a disruption of cell metabolism (59). Cellular proliferation and normal cell cycles are also affected, especially in lymphocytes (57–59). During these events, some cytokines might decrease or fluctuate (58). These different stimulatory and inhibitory cycles could have influenced the cytokine levels found in our analysis. Consequently, this could explain, in part, some of the heterogeneity found.

Some studies highlight the connections between cytokines (15). On one hand, this allowed us to suspect a combined effect of the retrieved cytokines on viral loads. Therefore, we included multiple cytokines (see Figures 2, 3) in some of the analyses. On the other hand, using meta-regression techniques and subgroup analysis, we investigated if unanticipated connections between molecules had influenced the results. In the comparisons between cytokine levels in healthy and HBV individuals, interleukin elimination led to no significant decrease in heterogeneity. Moreover, publication bias did not seem to alter the results obtained in Figure 2, as the funnel plot and Egger’s test indicated. We could say that, in this case, cytokine differences or potential connections between them did not influence the results of our study.

The meta-correlation analysis was the first large-scale assessment of 1,199 HBV patients and several different cytokines. Thus, we obtained a pooled correlation coefficient of 0.16 between cytokines and the levels of HBV-DNA, with moderate heterogeneity. We did not observe publication bias after trim-and-fill or the representation of the funnel plot. At first glimpse, according to the interpretation of Spearman correlation, the overall association between these two categories of elements was poor (60). Because of the differences between Spearman and Pearson correlations (61), we chose to avoid mixing articles with different correlations in one analysis. However, because of their small number, articles with Pearson correlation could not be included in a separate meta-analysis.

In subgroup analysis, we found a poor correlation between IL-6 and viral loads and a higher (fair) level of association between IL-10 and the levels of HBV-DNA. The results related to IL-6 had no significant heterogeneity (*I^2^* = 14%). Anabire et al. (62) contradict our findings. However, this study included a cohort of pregnant women with malaria and HBV, so multiple confounders could have influenced cytokine values. Other studies confirm our findings (22). We concluded that there is a clear tendency related to IL-6. On the other hand, our results on IL-10 expressed high heterogeneity, which we could not explain because of the small number of articles. Some authors contradict our results (22). However, they based their opinions on only 66 patients (by comparison to 97 from our subgroup analysis), among which they included acute HBV individuals (we analyzed only chronically infected people). Others claim a positive correlation between the levels of HBV-DNA and of HBV-RNA and a negative association between IL-10 and viral RNA in treated patients (63). However, treatment could have influenced their results (64). Therefore, we could not provide a definitive answer related to the correlation between IL-10 and the levels of HBV-DNA, but we could notice a tendency toward a positive result. Further controlled large-scale studies should conclude the debate.

Our meta-correlation tried to provide a thorough and accurate analysis. Articles that assessed IL-34, IL-12, and IL-35 were removed after meta-regression, with a significant decrease in heterogeneity. Our subgroup analysis in meta-correlation was also carefully inspected. When analyzed together, IL-10 and IL-9 led to the highest pooled correlation of all the retrieved cytokines-0.52. Heterogeneity also varied among cytokines in subgroup analysis, ranging from no significant to moderate and even significant. Therefore, not every retrieved cytokine correlated equally with viral loads. Thus, taking all molecules in one single analysis provided an overall poor association that could not be applied to every element. For instance, IL-10 and IL-9 had higher correlation coefficients with the levels of HBV-DNA. Their combination had a lower heterogeneity than that of the IL-10 articles alone. Therefore, IL-10 and IL-9 might exert an influence not only on viremia but also on each other.

Literature shows that IL-10 increases and affects the activity of CD8^+^ T lymphocytes (65). The release of this cytokine is strongly influenced by stimuli from B lymphocytes (65, 66). Other authors associate IL-9 with mechanisms related to immune memory (67). Research also links this cytokine to hepatocellular formation because of a complex molecular system that involves a subset of T-helper lymphocytes (68). According to some authors, the same cells also produce IL-10 (48). The interaction between IL-10 and IL-9 is not uncommon for liver infections. It has also been described previously by Franco et al. in patients carrying *Schistosoma mansoni* (69). On the other hand, You et al. report a strong correlation between the levels of HBV-DNA and T lymphocytes in chronic HBV patients (14). This could explain the associations between the above-mentioned cytokines and the levels of HBV-DNA, but further studies are needed to find the exact pathways involved in their connection.

When we compared data from patients with several levels of viremia, we obtained different results. Overall, people with HBV-DNA levels higher than 2,000 IU/mL had higher cytokine levels than those with lower viral loads. However, the pattern was not the same when we compared patients with very high levels of HBV-DNA (more than 10,000,000 IU/mL) and those with lower viremia loads. Another inconvenience was that we could not perform these calculations for every cytokine. Therefore, these results could only be applied to IL-17 and IL-12. Furthermore, we could only compare cytokine levels for a small number of people with high viral loads. Therefore, more studies are needed to establish the exact molecular mechanisms encountered in patients with high levels of HBV-DNA.

IL-17 is a molecule released from a subset of T lymphocytes mainly connected to the progression toward fibrosis, according to some authors (70). IL-12 contributes to the differentiation of T-helper lymphocytes (71). A recent study has shown that achieving an undetectable level of HBV-DNA after therapy could modify the numbers and activity patterns of T lymphocytes in chronic HBV patients (72). Therefore, we hypothesize that different viremia loads could also be reflected in the secretory activity of T lymphocytes, which could cause fluctuations in the levels of cytokines connected to cellular proliferation, such as IL-12 and IL-17. However, this theory needs further study.

### 4.2 The importance of this meta-analysis

This study has multiple strengths. It is a thorough analysis of many different cytokines and HBV patients. As we have already mentioned, this research controlled various types of biases and performed the first systematic quest of the associations between viral loads and cytokines. We could show a clear tendency for IL-6 related to viral loads. The meta-correlation also highlighted a potential correlation between IL-10 and IL-9, presumed from the results of the included studies. Furthermore, from the funnel plot (Figure 4) and data retrieval, we observed the need for more confounder-controlled, large-scale studies conducted on multiple cytokines.

### 4.3 The limitations of the meta-analysis

The major limitation of our study is that, despite its large numbers of cytokines and patients included, the number of studies performed for each cytokine is low. This could have sometimes caused heterogeneity. Publication bias is also hard to interpret under these circumstances. However, this opens new ways for further research that could clarify unexplained heterogeneity in our study. Another problem is related to the variability of the methodologies applied for each cytokine article.

### 4.4 Future research perspectives

However, despite this, the meta-analysis opens new possibilities for further research because it underlines the potential correlation between some cytokines and viral loads. This summarization is important because it shows the current gaps in knowledge for some cytokine pathways (such as IL-10, IL-9, and IL-8) and emphasizes possible variations of cytokine levels connected to the levels of HBV-DNA. Finally, the meta-analysis gives the idea of novel research related to the association of IL-10, IL-9, and the HBV viral loads that could be helpful, in time, in finding a cure or a new prognostic biomarker. These possibilities arise from the fact that authors find IL-10 pathways as current targets for novel therapies (73) while others experiment with this cytokine in different prognostic algorithms (74).

## 5 Conclusion

This meta-analysis is the first quality-controlled and systematic study of the correlations between cytokines and viral loads. It is, from our knowledge, the first research on a large cohort of HBV patients collected from multiple studies.We observed, from the results obtained, a possible association between IL-9 and IL-10 and viral loads. The two cytokines might also influence each other. Therefore, IL-10 and IL-9 can bring a new perspective on prognostic assessment and treatment strategy. Thus, we foresee a new understanding of the cytokine influence on HBV replication. Future studies could emerge from our summarized analysis.

## Data availability statement

The original contributions presented in the study are included in the article/Supplementary material, further inquiries can be directed to the corresponding author.

## Author contributions

MM: Conceptualization, Data curation, Formal analysis, Methodology, Validation, Visualization, Writing – original draft, Writing – review & editing. IM: Data curation, Formal analysis, Writing – review & editing. IC: Formal analysis, Supervision, Validation, Writing – review & editing.

## References

[1] Younossi ZM Wong G Anstee QM Henry L. The global burden of liver disease. Clin Gastroenterol Hepatol. (2023) 21:1978–91. doi: 10.1016/j.cgh.2023.04.015 37121527

[2] Howell J Seaman C Wallace J Xiao Y Scott N Davies J . Pathway to global elimination of hepatitis B: HBV cure is just the first step. Hepatology. (2023) 78:976–90. doi: 10.1097/HEP.0000000000000430, PMID: 37125643 PMC10442143

[3] Tu T Budzinska MA Shackel NA Urban S. HBV DNA integration: molecular mechanisms and clinical implications. Viruses. (2017) 9:75. doi: 10.3390/v9040075, PMID: 28394272 PMC5408681

[4] Locatelli M Quivy J-P Chapus F Michelet M Fresquet J Maadadi S . HIRA supports hepatitis B virus Minichromosome establishment and transcriptional activity in infected hepatocytes. Cell Mol Gastroenterol Hepatol. (2022) 14:527–51. doi: 10.1016/j.jcmgh.2022.05.007, PMID: 35643233 PMC9304598

[5] Wong GL-H Wong VW-S Chan HL-Y. Virus and host testing to manage chronic hepatitis B. Clin Infect Dis. (2016) 62:S298–305. doi: 10.1093/cid/ciw024, PMID: 27190319 PMC4889896

[6] Mak L-Y Hui RW-H Fung J Seto WK Yuen M-F. The role of different viral biomarkers on the management of chronic hepatitis B. Clin Mol Hepatol. (2023) 29:263–76. doi: 10.3350/cmh.2022.0448, PMID: 36655304 PMC10121282

[7] Varghese N Majeed A Nyalakonda S Boortalary T Halegoua-DeMarzio D Hann H-W. Review of related factors for persistent risk of hepatitis B virus-associated hepatocellular carcinoma. Cancers. (2024) 16:777. doi: 10.3390/cancers16040777 38398168 PMC10887172

[8] Bousali M Karamitros T. Hepatitis B virus integration into transcriptionally active loci and HBV-associated hepatocellular carcinoma. Microorganisms. (2022) 10:253. doi: 10.3390/microorganisms10020253, PMID: 35208708 PMC8879149

[9] Qian Z Liang J Huang R Song W Ying J Bi X . HBV integrations reshaping genomic structures promote hepatocellular carcinoma. Gut. (2024) 73:gutjnl-2023-330414–1182. doi: 10.1136/gutjnl-2023-330414, PMID: 38395437 PMC11187386

[10] Allweiss L Testoni B Yu M Lucifora J Ko C Qu B . Quantification of the hepatitis B virus cccDNA: evidence-based guidelines for monitoring the key obstacle of HBV cure. Gut. (2023) 72:972–83. doi: 10.1136/gutjnl-2022-328380, PMID: 36707234 PMC10086470

[11] Tunçbilek S. Relationship between cytokine gene polymorphisms and chronic hepatitis B virus infection. World J Gastroenterol. (2014) 20:6226–35. doi: 10.3748/wjg.v20.i20.6226, PMID: 24876743 PMC4033460

[12] Zhong S Zhang T Tang L Li Y. Cytokines and chemokines in HBV infection. Front Mol Biosci. (2021) 8:805625. doi: 10.3389/fmolb.2021.805625, PMID: 34926586 PMC8674621

[13] Wautier J-L Wautier M-P. Pro- and anti-inflammatory prostaglandins and cytokines in humans: a mini review. Int J Mol Sci. (2023) 24:9647. doi: 10.3390/ijms24119647, PMID: 37298597 PMC10253712

[14] You J Sriplung H Geater A Chongsuvivatwong V Zhuang L Chen H-Y . Effect of viral load on T-lymphocyte failure in patients with chronic hepatitis B. World J Gastroenterol. (2008) 14:1112–9. doi: 10.3748/wjg.14.1112, PMID: 18286696 PMC2689417

[15] Cui A Huang T Li S Ma A Pérez JL Sander C . Dictionary of immune responses to cytokines at single-cell resolution. Nature. (2024) 625:377–84. doi: 10.1038/s41586-023-06816-9, PMID: 38057668 PMC10781646

[16] Janovec V Hodek J Clarova K Hofman T Dostalik P Fronek J . Toll-like receptor dual-acting agonists are potent inducers of PBMC-produced cytokines that inhibit hepatitis B virus production in primary human hepatocytes. Sci Rep. (2020) 10:12767. doi: 10.1038/s41598-020-69614-7, PMID: 32728070 PMC7392756

[17] Jamaludeen N Lehmann J Beyer C Vogel K Pierau M Brunner-Weinzierl M . Assessment of immune status using inexpensive cytokines: a literature review and learning approaches. Sensors. (2022) 22:9785. doi: 10.3390/s22249785, PMID: 36560154 PMC9786078

[18] Wu Z-B Zheng Y-B Wang K Mo Z-S Zhen X Yan Y . Plasma Interleukin-6 level: a potential prognostic indicator of emergent HBV-associated ACLF. Can J Gastroenterol Hepatol. (2021) 2021:5545181–7. doi: 10.1155/2021/5545181, PMID: 34805027 PMC8601797

[19] Chua C Salimzadeh L Ma AT Adeyi OA Seo H Boukhaled GM . IL-2 produced by HBV-specific T cells as a biomarker of viral control and predictor of response to PD-1 therapy across clinical phases of chronic hepatitis B. Hepatol Commun. (2023) 7:e0337. doi: 10.1097/HC9.0000000000000337, PMID: 38055623 PMC10984660

[20] Wang W-X Jia R Jin X-Y Li X Zhou S-N Zhang X-N . Serum cytokine change profile associated with HBsAg loss during combination therapy with PEG-IFN-α in NAs-suppressed chronic hepatitis B patients. Front Immunol. (2023) 14:1121778. doi: 10.3389/fimmu.2023.1121778, PMID: 36756119 PMC9899895

[21] Hsu Y-C Tseng C-H Kao J-H. Safety considerations for withdrawal of nucleos(t)ide analogues in patients with chronic hepatitis B: first, do no harm. Clin Mol Hepatol. (2023) 29:869–90. doi: 10.3350/cmh.2022.0420, PMID: 36916171 PMC10577354

[22] Ribeiro CR d A Beghini DG Lemos AS Martinelli KG de Mello V d M de Almeida NAA . Cytokines profile in patients with acute and chronic hepatitis B infection. Microbiol Immunol. (2022) 66:31–9. doi: 10.1111/1348-0421.12947, PMID: 34647645

[23] Page MJ Moher D Bossuyt PM Boutron I Hoffmann TC Mulrow CD . PRISMA 2020 explanation and elaboration: updated guidance and exemplars for reporting systematic reviews. BMJ. (2021) 372:n160. doi: 10.1136/bmj.n160, PMID: 33781993 PMC8005925

[24] *PlotDigitizer* (2024). Available at: https://plotdigitizer.com (Accessed March 13, 2024).

[25] *ZOTERO* (2024). Available at: https://zotero.org (Accessed February 23, 2023).

[26] Wells GA Shea B O’Connell D Pereson J Welch V Losos M . The Newcastle-Ottawa scale (NOS) for assessing the quality of nonrandomized studies in meta-analysis (2024). Available at: https://www.ohri.ca/oxford (Accessed March 1, 2024).

[27] Herzog R Álvarez-Pasquin MJ Díaz C Del Barrio JL Estrada JM Gil Á. Are healthcare workers’ intentions to vaccinate related to their knowledge, beliefs and attitudes? A systematic review. BMC Public Health. (2013) 13:154. doi: 10.1186/1471-2458-13-154, PMID: 23421987 PMC3602084

[28] Waweru P Gatimu SM. Stroke epidemiology, care, and outcomes in Kenya: a scoping review. Front Neurol. (2021) 12:785607. doi: 10.3389/fneur.2021.785607, PMID: 34975737 PMC8716633

[29] Kalaycioglu I Rioux B Briard JN Nehme A Touma L Dansereau B . Inter-rater reliability of risk of bias tools for non-randomized studies. Syst Rev. (2023) 12:227. doi: 10.1186/s13643-023-02389-w, PMID: 38057883 PMC10702000

[30] R Core Team. R: a language and environment for statistical computing. Vienna, Austria: R Foundation for Statistical Computing (2022).

[31] Borenstein M Hedges LV Higgins JPT Rothstein HR. A basic introduction to fixed-effect and random-effects models for meta-analysis. Res Synth Methods. (2010) 1:97–111. doi: 10.1002/jrsm.12, PMID: 26061376

[32] Luo D Wan X Liu J Tong T. Optimally estimating the sample mean from the sample size, median, mid-range, and/or mid-quartile range. Stat Methods Med Res. (2018) 27:1785–805. doi: 10.1177/0962280216669183, PMID: 27683581

[33] Shi J Luo D Weng H Zeng X-T Lin L Chu H . Optimally estimating the sample standard deviation from the five-number summary. Res Synth Methods. (2020) 11:641–54. doi: 10.1002/jrsm.1429 32562361

[34] Cooper H Hedges LV Valentine JC. The handbook of research synthesis and Metaanalysis. 2nd ed. New York: Russell Sage Foundation (2009).

[35] Higgins JPT Thompson SG Deeks JJ Altman DG. Measuring inconsistency in meta-analyses. BMJ. (2003) 327:557–60. doi: 10.1136/bmj.327.7414.557, PMID: 12958120 PMC192859

[36] Guddat C Grouven U Bender R Skipka G. A note on the graphical presentation of prediction intervals in random-effects meta-analyses. Syst Rev. (2012) 1:34. doi: 10.1186/2046-4053-1-34, PMID: 22839660 PMC3552946

[37] Sterne JA Egger M. Funnel plots for detecting bias in meta-analysis: guidelines on choice of axis. J Clin Epidemiol. (2001) 54:1046–55. doi: 10.1016/s0895-4356(01)00377-8 11576817

[38] Egger M Davey Smith G Schneider M Minder C. Bias in meta-analysis detected by a simple, graphical test. BMJ. (1997) 315:629–34. doi: 10.1136/bmj.315.7109.629, PMID: 9310563 PMC2127453

[39] Egger M Smith GD Phillips AN. Meta-analysis: principles and procedures. BMJ. (1997) 315:1533–7. doi: 10.1136/bmj.315.7121.1533, PMID: 9432252 PMC2127925

[40] Park Y Park Y Han K Kim H. Serum cytokine levels in patients with chronic hepatitis B according to lamivudine therapy. J Clin Lab Anal. (2011) 25:414–21. doi: 10.1002/jcla.20495, PMID: 22086795 PMC6647680

[41] Elbrolosy AM Elabd NS ElGedawy GA Abozeid M Abdelkreem M Montaser B . Toll- like receptor 2 polymorphism and IL-6 profile in relation to disease progression in chronic HBV infection: a case control study in Egyptian patients. Clin Exper Med. (2023) 23:117–29. doi: 10.1007/s10238-022-00792-6, PMID: 35119591 PMC9939497

[42] Li C Ji H Cai Y Ayana DA Lv P Liu M . Serum Interleukin-37 concentrations and HBeAg seroconversion in chronic HBV patients during Telbivudine treatment. J Interf Cytokine Res. (2013) 33:612–8. doi: 10.1089/jir.2013.0001, PMID: 23697556 PMC3793653

[43] Li J Ren W Ma W Zhang J Shi J Qin C. Interleukin-21 responses in patients with chronic hepatitis B. J Interf Cytokine Res. (2015) 35:134–42. doi: 10.1089/jir.2013.0119, PMID: 25243706 PMC4312796

[44] Zhong H Xibing G Yaping D Zheng W Decai F Xiaoye G . Interleukin-7 in patients with chronic hepatitis B may have effect on T follicular helper cells and specific cellular immunity. Hepat Mon. (2016) 16:e36068. doi: 10.5812/hepatmon.36068, PMID: 27822258 PMC5091030

[45] Cheng S-T Tang H Ren J-H Chen X Huang A-L Chen J. Interleukin-34 inhibits hepatitis B virus replication *in vitro* and *in vivo*. PLoS One. (2017) 12:e0179605. doi: 10.1371/journal.pone.0179605, PMID: 28614380 PMC5470710

[46] Shao X Ma J Jia S Yang L Wang W Jin Z. Interleukin-35 suppresses antiviral immune response in chronic hepatitis B virus infection. Front Cell Infect Microbiol. (2017) 7:472. doi: 10.3389/fcimb.2017.00472, PMID: 29181338 PMC5693856

[47] Cheng S-T Yuan D Liu Y Huang Y Chen X Yu H-B . Interleukin-35 level is reduced in patients with chronic hepatitis B virus infection. Int J Med Sci. (2018) 15:188–94. doi: 10.7150/ijms.21957, PMID: 29333103 PMC5765732

[48] Cui M Lv Y Lu J Zhang W Duan Y Huang Y . Decreased frequency of circulating Th9 cells in patients with chronic hepatitis B infection. J Clin Lab Anal. (2018) 32:e22246. doi: 10.1002/jcla.22246, PMID: 28481430 PMC6817209

[49] Luo L Jiang L Tian Z Zhang X. The serum interleukin-26 level is a potential biomarker for chronical hepatitis B. Medicine. (2020) 99:e18462. doi: 10.1097/MD.0000000000018462, PMID: 31895778 PMC6946192

[50] Yang J Guo R Yan D Lu H Zhang H Ye P . Plasma level of ADAMTS13 or IL-12 as an indicator of HBeAg seroconversion in chronic hepatitis B patients undergoing m-ETV treatment. Front Cell Infect Microbiol. (2020) 10:335. doi: 10.3389/fcimb.2020.00335, PMID: 32793509 PMC7393286

[51] Ren S Wang W Lu J Wang K Ma L Zheng Y . Effect of the change in antiviral therapy indication on identifying significant liver injury among chronic hepatitis B virus infections in the grey zone. Front Immunol. (2022) 13:1035923. doi: 10.3389/fimmu.2022.1035923, PMID: 36389814 PMC9647141

[52] Zhou F Xiong H Zhen S Chen A Huang M Luo Y. Serum levels of IL-12, IL-18, and IL-21 are indicators of viral load in patients chronically infected with HBV. Braz J Med Biol Res. (2022) 55:e12320. doi: 10.1590/1414-431X2022e12320 36383803 PMC9668081

[53] Okuhara S Umemura T Joshita S Shibata S Kimura T Morita S . Serum levels of interleukin-22 and hepatitis B core-related antigen are associated with treatment response to entecavir therapy in chronic hepatitis B. Hepatol Res. (2014) 44:E172–80. doi: 10.1111/hepr.12287, PMID: 24308754

[54] Metanat M Alijani E Ansari-Moghaddam A Bahrehmand F Khalili M Arbabi N . The relationship between serum IL-17 level and viral load in chronic hepatitis B. Arch Clin Infect Dis. (2019) 14:e68172. doi: 10.5812/archcid.68172

[55] Wiegand SB Beggel B Wranke A Aliabadi E Jaroszewicz J Xu C-J . Soluble immune markers in the different phases of chronic hepatitis B virus infection. Sci Rep. (2019) 9:14118. doi: 10.1038/s41598-019-50729-5, PMID: 31575964 PMC6773856

[56] Peters MG Yuen M-F Terrault N Fry J Lampertico P Gane E . Chronic hepatitis B finite treatment: similar and different concerns with new drug classes. Clin Infect Dis. (2024) 78:983–90. doi: 10.1093/cid/ciad506, PMID: 37633256 PMC11006103

[57] Stella L Santopaolo F Gasbarrini A Pompili M Ponziani FR. Viral hepatitis and hepatocellular carcinoma: from molecular pathways to the role of clinical surveillance and antiviral treatment. World J Gastroenterol. (2022) 28:2251–81. doi: 10.3748/wjg.v28.i21.2251, PMID: 35800182 PMC9185215

[58] Torresi J Tran BM Christiansen D Earnest-Silveira L Schwab RHM Vincan E. HBV-related hepatocarcinogenesis: the role of signalling pathways and innovative ex vivo research models. BMC Cancer. (2019) 19:707. doi: 10.1186/s12885-019-5916-6, PMID: 31319796 PMC6637598

[59] Li Y Ou JJ. Regulation of mitochondrial metabolism by hepatitis B virus. Viruses. (2023) 15:2359. doi: 10.3390/v15122359, PMID: 38140600 PMC10747323

[60] Chan YH. Biostatistics 104: correlational analysis. Singapore Med J. (2003) 44:614–9. PMID: 14770254

[61] Artusi R Verderio P Marubini E. Bravais-Pearson and Spearman correlation coefficients: meaning, test of hypothesis and confidence interval. Int J Biol Markers. (2002) 17:148–51. doi: 10.1177/172460080201700213, PMID: 12113584

[62] Anabire NG Aryee PA Abdul-Karim A Quaye O Awandare GA Helegbe GK. Impact of malaria and hepatitis B co-infection on clinical and cytokine profiles among pregnant women. PLoS One. (2019) 14:215550. doi: 10.1371/journal.pone.0215550, PMID: 31002731 PMC6474591

[63] Zhang Q Huang H Sun A Liu C Wang Z Shi F . Change of cytokines in chronic hepatitis B patients and HBeAg are positively correlated with HBV RNA, based on real-world study. J Clin Transl Hepatol. (2022) 10:390–7. doi: 10.14218/JCTH.2021.00160, PMID: 35836760 PMC9240249

[64] Narayanan S Au VB Khakpoor A Yan C Ahl PJ Kaliaperumal N . Bayesian analysis of cytokines and chemokine identifies immune pathways of HBsAg loss during chronic hepatitis B treatment. Sci Rep. (2021) 11:7455. doi: 10.1038/s41598-021-86836-5, PMID: 33811250 PMC8018960

[65] Rojas JM Avia M Martín V Sevilla N. IL-10: a multifunctional cytokine in viral infections. J Immunol Res. (2017) 2017:e6104054:1–14. doi: 10.1155/2017/6104054, PMID: 28316998 PMC5337865

[66] de Gruijter NM Jebson B Rosser EC. Cytokine production by human B cells: role in health and autoimmune disease. Clin Exp Immunol. (2022) 210:253–62. doi: 10.1093/cei/uxac090, PMID: 36179248 PMC9985175

[67] Choi J Crotty S Choi YS. Cytokines in follicular helper T cell biology in physiologic and pathologic conditions. Immune Netw. (2024) 24:e8. doi: 10.4110/in.2024.24.e8, PMID: 38455461 PMC10917579

[68] Chen T Guo J Cai Z Li B Sun L Shen Y . Th9 cell differentiation and its dual effects in tumor development. Front Immunol. (2020) 11:1026. doi: 10.3389/fimmu.2020.01026, PMID: 32508847 PMC7251969

[69] Franco KGS de Amorim FJR Santos MA Rollemberg CVV de Oliveira FA França AVC . Association of IL-9, IL-10, and IL-17 cytokines with hepatic fibrosis in human *Schistosoma mansoni* infection. Front Immunol. (2021) 12:779534. doi: 10.3389/fimmu.2021.779534, PMID: 34970264 PMC8712476

[70] Paquissi FC. Immunity and Fibrogenesis: the role of Th17/IL-17 Axis in HBV and HCV-induced chronic hepatitis and progression to cirrhosis. Front Immunol. (2017) 8:1195. doi: 10.3389/fimmu.2017.01195, PMID: 29033929 PMC5626935

[71] Dong L He Y Cao Y Wang Y Jia A Wang Y . Functional differentiation and regulation of follicular T helper cells in inflammation and autoimmunity. Immunology. (2021) 163:19–32. doi: 10.1111/imm.13282, PMID: 33128768 PMC8044332

[72] Narmada BC Khakpoor A Shirgaonkar N Narayanan S Aw PPK Singh M . Single cell landscape of functionally cured chronic hepatitis B patients reveals activation of innate and altered CD4-CTL-driven adaptive immunity. J Hepatol. (2024) 81:42–61. doi: 10.1016/j.jhep.2024.02.017, PMID: 38423478

[73] Wang D Fu B Wei H. Advances in immunotherapy for hepatitis B. Pathogens. (2022) 11:1116. doi: 10.3390/pathogens11101116, PMID: 36297173 PMC9612046

[74] Saxena R Chawla YK Verma I Kaur J. Association of interleukin-10 with hepatitis B virus (HBV) mediated disease progression in Indian population. Indian J Med Res. (2014) 139:737–45. PMID: 25027084 PMC4140039

