# Supplementary material for: Extended analysis on peripheral blood cytokines correlated with hepatitis B virus viral load in chronically infected patients – a systematic review and meta-analysis
Source: Front Med (Lausanne). 2024 Jul 31;11:1429926. doi: 10.3389/fmed.2024.1429926 (PMC11325457; doi:10.3389/fmed.2024.1429926)
Supplement: Supplementary file 1 [file Table_1.docx]

Supplementary Table S1. Study protocol synthesis

| Review questions | Are potential correlations between cytokines and HBV-DNA level? Are there any differences between cytokine levels in chronically infected HBV patients by comparison to healthy subjects? Are there any differences between cytokine levels in different categories of chronic HBV patients (individuals with low, moderate and high viral loads)? |
| --- | --- |
| Searches | PUBMED, WEB OF SCIENCE, SCOPUS, and Cochrane Library were the four databases used. |
| Inclusion criteria | Correlations between cytokines and viral load.  Cytokine values for different viremia levels and for healthy subjects.  English-written articles with full-text availability. |
| Exclusion criteria | Inclusion criteria not fulfilled.  Abstracts, pre-prints, proceedings, conference recordings, reviews, meta-analyses, systematic reviews, book chapters, surveys, editorials, notes, letters, and commentaries.  Retracted or duplicate papers. |
| Population, exposures, comparators, outcomes | We searched every cytokine quantification present in chronically infected HBV patients with different levels of HBV-DNA (low, moderate and high) and in healthy subjects. We also extracted data related to the correlation between cytokines and viremia. |
| Data extraction | MM and IM independently collected, assessed and extracted data. They analyzed all entries from inception until March 7th, 2024, according to the flow diagram provided in **Figure 1**. Discussion and third-party evaluation by IC solved every difference in opinion. Software used: PlotDigitizer (23) and ZOTERO (24). |
| Risk of bias and quality assessment | Tools: Newcastle-Ottawa Quality Assessment Scale (NOS) (25), modified NOS scale (26, 27). |
| Statistical analysis | Software: R 4.2.2 Software (R Foundation for Statistical Computing, Vienna, Austria) for statistical analysis (29).  Method: Random-effect method with an inverse variance weighting model, standardized mean difference calculation, Fisher’s z-transformed correlations  Heterogeneity assessment: *I^2^* test, forest plot, funnel plot, Egger test, trim-and-fill method, subgroup analysis, meta-regression |

HBV-hepatitis B virus
