# Supplementary material for: Extended analysis on peripheral blood cytokines correlated with hepatitis B virus viral load in chronically infected patients – a systematic review and meta-analysis
Source: Front Med (Lausanne). 2024 Jul 31;11:1429926. doi: 10.3389/fmed.2024.1429926 (PMC11325457; doi:10.3389/fmed.2024.1429926)
Supplement: Supplementary file 2 [file Table_2.docx]

Supplementary Table S2. General search terms

| Main search terms | ((chronic hepatitis B) OR (chronic HBV infection) OR (viral hepatitis B) OR (HBV infection) OR (HBV) OR (VHB) OR (hepatitis B virus)) AND ((low viremia) OR (low HBV DNA) OR (low viral DNA) OR (HBV DNA) OR (high viremia) OR (high HBV DNA) OR (high viral DNA)) AND ((Il) OR (IL) OR (interleukin) OR (cytokine) OR (cytokine assay) OR (interleukin assay)) |
| --- | --- |

HBV-hepatitis B virus, IL or Il-interleukin, VHB- viral hepatitis B.
