# Supplementary material for: Extended analysis on peripheral blood cytokines correlated with hepatitis B virus viral load in chronically infected patients – a systematic review and meta-analysis
Source: Front Med (Lausanne). 2024 Jul 31;11:1429926. doi: 10.3389/fmed.2024.1429926 (PMC11325457; doi:10.3389/fmed.2024.1429926)
Supplement: Supplementary file 3 [file Table_3.docx]

Supplementary Table S3. Cytokine levels among different study groups

| **Study**  **(Author, Year)** | **Country** | **Study type** | **Groups of interest** | **Age-mean (years)** | **Method for cytokine detection** | **Cytokine studied** | **Cytokine levels (pg/mL)** | | | | | | **NOS** |
| --- | --- | --- | --- | --- | --- | --- | --- | --- | --- | --- | --- | --- | --- |
| Li et al., 2013 (42) | China | Cohort | 40 CHB patients (baseline measurements- HBV-DNA> 10000 IU/mL and after telbivudine treatment- HBV-DNA< 10000 IU/mL) | 39.31± 9.48 | Cytokine bead array (for serum IL-2 and IL-10)  Elisa (for serum IL-37) | IL-2 | Baseline:6.96± 2.72 | | After telbivudine:12.62± 4.07 | | HC: 12.04± 3.2 | | 8 |
|  |  |  |  |  |  | IL-10 | Baseline:6.81± 1.88 | | After telbivudine: 7.61± 1.99 | | HC: 13.18± 3.55 | |  |
|  |  |  | 30 HC | 35.7± 8.32 |  | IL-37 | Baseline:76.91± 63.11 | | After telbivudine: 37.86± 25.34 | | HC: 27.37± 8.6 | |  |
| Metanat et al., 2019 (54) | Iran | Cross-sectional | 74 CHB patients with HBV-DNA<2000 IU/mL | NA | Elisa (for serum IL-17) | IL-17 | HBV-DNA< 2000 IU/ml group: 30.66±103.65 | | HBV-DNA between 2000 IU/ml and 10^7^ IU/ml group: 26.87± 91.68 | | HBV-DNA> 10^7^ IU/ml: 24.42± 75.5 | | 7* |
|  |  |  | 53 CHB patients with HBV-DNA between 2000 IU/mL and 10^7^ IU/mL |  |  |  |  |  |  |  |  |  |  |
|  |  |  | 16 CHB patients with HBV-DNA > 10^7^ IU/mL |  |  |  |  |  |  |  |  |  |  |
| Wiegand et al., 2019 (55) | Germany | Cross-sectional | 88 HBV patients with chronic infection, HBeAg negative, and HBV-DNA< 2000 IU/mL (Group 1) | 40.94± 11.61 | Luminex (for serum cytokines) | IL-17  TGF-β1  TGF-β2  TGF-β3  TNF-α  IL-12  IL-16 | Group 1:  IL-17: 1.49±2.02; TGF-β1: 29279.02±33675.5; TGF-β2: 4706.15± 1762.2;TGF-β3: 1493.4±1492.1; TNF-α: 1.86± 2.67; IL-12: 0.65± 1.26; IL-16: 88.2±127.7 | | Group 2:  IL-17: 2.46±3.73; TGF-β1: 56521.1±79073.8; TGF-β2: 5144.8± 1989.3;TGF-β3:1804.1±1681.2; TNF-α:16.22± 32.6; IL-12: 2.63± 5.22; IL-16: 291.3± 545.8 | | Group 3 :  IL-17:1.03± 1.48; TGF-β1: 23808.6± 21502.2; TGF-β2: 3772.5± 2215;TGF-β3: 1212.8± 1190.5; TNF-α: 2.5± 4.04; IL-12: 0.7± 1.31; IL-16: 69.11± 88.94 | | 7* |
|  |  |  | 63 HBV patients with chronic hepatitis, HBeAg negative, and HBV-DNA> 2000 IU/mL (Group 2) | 41.43± 9.17 |  |  |  |  |  |  |  |  |  |
|  |  |  | 48 HBV patients with chronic infection, HBeAg negative, and HBV-DNA between 2000 IU/ml and 20000 IU/mL (Group 3) | 30.46± 9.98 |  |  |  |  |  |  |  |  |  |
| Luo et al., 2020 (49) | China | Cohort | 30 CHB patients (baseline measurements-HBV-DNA> 100000 IU/mL and after telbivudine treatment- HBV-DNA< 100000 IU/mL) | NA | Elisa (for serum IL-26 and IL-17) | IL-26 | Baseline: 5050± 440 | | After telbivudine: 2850± 300 | | HC: 790± 170 | | 9 |
|  |  |  | 28 HC |  |  | IL-17 | Baseline: 1250± 810 | | - | | HC: 5190± 490 | |  |
| Zhou et al., 2022 (52) | China | Case-control | 48 CHB patients- Group 1: (HBV-DNA between 1000 IU/ml and 10000 IU/mL) | 46.33± 13.13 | Elisa (for serum IL-12, IL-18, and IL-21) | IL-12 | - | Group 2 : 90.27± 59.96 | | Group 3 : 59.66± 34.28 | | HC : 29.85± 6.48 | 8 |
|  |  |  | 47 CHB patients- Group 2: (HBV-DNA between 100000 IU/ml and 1000000 IU/mL) | 44.27± 14.43 |  | IL-18 | Group 1: 266.9± 203.53 | Group 2: 364.83± 233.87 | | Group 3: 240.96± 134.06 | | HC: 78.75± 4.89 |  |
|  |  |  | 47 CHB patients- Group 3: (HBV-DNA between 10000000 IU/mL and 100000000 IU/mL) | 36.46± 12.01 |  | IL-21 | Group 1: 1.03± 1.35 | Group 2: 3.21± 5.8 | | - | | HC: 0.69± 0.42 |  |
|  |  |  | 20 HC | 42.53± 13.58 |  |  |  |  |  |  |  |  |  |
| Elbrolosy et al., 2023 (41) | Egypt | Case-control | 65 patients with inactive chronic HBV infection- HBV-DNA< 2000 IU/mL (Group 1) | 52.5± 11.8 | Elisa (for serum IL-6) | IL-6 | Group 1: 41.3± 10.7 | | Group 2: 74.9± 23.8 | | HC: 15± 3.3 | | 8 |
|  |  |  | 62 patients with active CHB – HBV-DNA> 2000 IU/mL (Group 2) | 55.3± 9.4 |  |  |  |  |  |  |  |  |  |
|  |  |  | 60 HC | 55.6± 10.4 |  |  |  |  |  |  |  |  |  |

CHB- chronic hepatitis B; HBV-hepatitis B virus; HC-healthy controls; HBV-DNA- level of HBV-DNA; IL-interleukin; TGF-β1,2,3- transforming growth factor beta 1, 2, 3; TNF-α- tumor necrosis factor alpha; NOS-NewCastle-Ottawa scale; all values are presented as mean± standard deviation.

*studies that required adapted NOS (26,27)
